# Early diagnosis of inborn errors of immunity in childhood: from clinical suspicion to molecular diagnosis

**DOI:** 10.36416/1806-3756/e20260490

**Published:** 2026-11-09

**Authors:** Amanda Bendorovicz Trevisan Dorneles, Laura Zaffari Leal, Maria Paula De Carli Hanel, Leonardo Araújo Pinto

**Affiliations:** 1. Grupo de Pesquisa em Epidemiologia e Genética das Doenças Respiratórias da Infância, Pontifícia Universidade Católica do Rio Grande do Sul - PUCRS - Porto Alegre (RS) Brasil.; 2. Programa de Pós-Graduação em Medicina - Pediatria, Escola de Medicina, Pontifícia Universidade Católica do Rio Grande do Sul - PUCRS - Porto Alegre (RS) Brasil.

## INTRODUCTION

Inborn errors of immunity (IEI), historically called primary immunodeficiencies, are a heterogeneous group of congenital diseases that primarily impair the function of the immune system. Advances in genetic knowledge have profoundly changed this concept: current guidelines recognize defects in approximately 500 genes associated with IEI, encompassing phenotypes that go beyond susceptibility to infection and include autoimmunity, autoinflammation, severe atopy, bone marrow failure, and malignancies.^1^ Despite these advances, diagnosis often remains delayed, with recent data indicating a mean interval of approximately four years between symptom onset and recognition of the disease.^2^ This delay favors cumulative morbidity and the development of organ damage before appropriate treatment is instituted. In this context, the principle that “time is lung” encapsulates the goal of early diagnosis: recognizing IEI before recurrent infections cause irreversible structural sequelae.

## PRESCHOOL AND IMMUNODEFICIENCY

In childhood, the main clinical challenge is to distinguish ordinary infectious exposure, common in children who attend daycare, from a pattern of immune failure. The frequency of infections alone is poorly informative: in immunocompetent children, episodes tend to be viral, self-limited, and quick to resolve; in IEI, what stands out is unusual recurrence, severity, and duration, inadequate response to treatment, the need for prolonged or intravenous antimicrobials, and infections with opportunistic agents.^1^ ^,^ ^2^

As a screening tool, the use of the following warning signs is suggested: 1. four or more ear infections per year; 2. two or more serious sinus infections per year; 3. antibiotics for two or more months with little effect; 4. two or more pneumonias per year; 5. growth failure; 6. recurrent deep abscesses; 7. persistent candidiasis; 8. two or more deep infections, including sepsis; and 9. family history of immunodeficiency. These signs are practically useful for screening but have important limitations: they should not be used in isolation to exclude IEI, and their sensitivity varies by age group, being lower in infants. In addition, chronic diarrhea, parental consanguinity, and a family history of tuberculosis have been proposed as additional warning signs.^2^

The contemporary conception of IEI further broadens the clinical manifestations, including noninfectious presentations, such as immune dysregulation, autoimmune cytopenias, and severe or refractory allergy, which may be the first expression of an IEI in some diseases, so the absence of the classic infectious phenotype does not rule out the diagnosis.^1^

## NEWBORN SCREENING: EARLY DIAGNOSIS

Newborn screening makes it possible to identify IEI before symptoms appear and to prevent sequelae and mortality. The expanded heel-prick test (*Teste do Pezinho*) assesses, by real-time PCR on filter paper, two biomarkers of lymphocyte maturation: T-cell receptor excision circles (TREC), the gold standard for severe combined immunodeficiencies (SCID) and congenital athymia, which measures recent thymic emigrant naïve T lymphocytes; and Kappa-deleting recombination excision circles (KREC), which reflects B-lymphocyte maturation and allows the identification of agammaglobulinemias.^1^ ^,^ ^3^ The second screening approach is based on clinical suspicion grounded in the warning signs already addressed above. 

The rationale for this strategy is straightforward: when hematopoietic stem cell transplantation is performed before 3.5 months of age and before the onset of active infection, survival in SCID reaches 90-94%, a value far higher than that seen in children diagnosed only after clinical manifestation, in addition to making it possible to avoid contraindicated live-virus vaccines and to reduce hospitalizations.^3^

In Brazil, screening was incorporated into the National Neonatal Screening Program by Federal Law No. 14,154/2021, but its implementation remains uneven across states, restricted mainly to pioneering programs, which keeps the prevalence of late diagnosis high.^2^

## MAIN ETIOLOGIC DIAGNOSES

The International Union of Immunological Societies divides IEI into nine pathophysiological categories.^1^ Epidemiologically, however, antibody deficiencies (such as selective IgA deficiency and CVID) and syndromic forms (such as DiGeorge syndrome) predominate, and these frequently trigger the clinical warning signs. Critical cases, such as SCID, require presymptomatic detection through newborn screening.^3^ Currently, definitive etiologic diagnosis in all categories is driven by next-generation sequencing.^4^ Early elucidation is vital because delayed recognition results in recurrent infections and irreversible damage, with bronchiectasis as the central sequela.^5^

[Table t1a]

**Table 1A t1a:** Stepwise diagnosis recommendations.

Phase	Tool	Ideal timing	Prognostic impact	Ref.
Presymptomatic	TREC/KREC (heel-prick test)	Newborn period	Very high survival (90-94%) with early HSCT (<3.5 months) performed before active infections	[3]
Clinical suspicion (PHC)	JMF warning signs + CBC + Ig	First pediatric visit	Speeds up and guides referral, reducing exposure to severe infections	[1,2]
Specialized immunologic confirmation	Immunophenotyping, lymphocyte function	Weeks after suspicion	Allows the introduction of life-saving supportive therapies, stabilizing the clinical picture	[1]
Molecular confirmation	NGS (panel/exome)	After laboratory suspicion	Changes management in up to 38% and enables genetic counseling in 53%	[4]
Late diagnosis	-	Usually 2-4 years	High morbidity and mortality associated with bronchiectasis	[5]

[Table t1b]

**Table 1B t1b:** Main etiologic diagnosis.

Diagnosis	Main manifestations	Characteristics	Tests	Ref.
SCID	Severe infections from the first months of life	Severe T-cell lymphopenia; hypoplastic/absent thymus	TREC low (heel-prick test)	[1,3]
			Immunophenotyping: low T cells	
X-linked agammaglobulinemia (Bruton)	Bacterial sinopulmonary infections from 6 months; reduced/absent lymphoid tissue; almost exclusively boys	BTK mutation blocking B-cell maturation in bone marrow; near-absent circulating B cells	KREC low (heel-prick test)	[1,3]
			Serum Ig very low/absent	
			BTK genetic test	
Selective IgA deficiency	Mostly asymptomatic; if symptomatic, mild recurrent respiratory/gastrointestinal infections. Association with celiac disease	Most prevalent primary immunodeficiency. Very low serum IgA with normal IgG and IgM	Serum IgA low; IgG/IgM normal	[1]
			Repeat after age 4	
CVID (common variable immunodeficiency)	Recurrent bacterial sinopulmonary infections. Autoimmunity, bronchiectasis, lymphoproliferation, increased lymphoma risk	Classic hypogammaglobulinemia (low IgG with low IgA and/or IgM). Poor vaccine response	Serum IgG low + IgA and/or IgM low	[1]
			Poor polysaccharide vaccine response	
			Exclude secondary causes	
Selective IgG subclass deficiency	Recurrent respiratory infections (sinusitis, otitis, pneumonia) or asymptomatic	Normal total IgG with reduction of one or more subclasses (IgG1-IgG4). Clinical significance often controversial	IgG subclass(es) low Vaccine response assessment	[1]

## STEPWISE DIAGNOSTIC ALGORITHM

The investigation should follow a stepwise logic, according to the level of care available. At Level 1, in primary health care, the presence of warning signs prompts a complete blood count, HIV serology to exclude a secondary cause, and measurement of serum immunoglobulins (IgA, IgG, IgM, and IgE). Abnormal findings lead to referral to Level 2, where the specialist deepens the investigation with immunophenotyping and assessment of lymphocyte function, tests capable of identifying specific cellular and humoral defects. Finally, Level 3, at a referral center, confirms the diagnosis through genetic testing, defining the molecular defect and guiding definitive treatment. This logic avoids both excessive investigation in children with a trivial infectious picture and delay in cases that require urgent intervention, optimizing the use of specialized resources. Molecular confirmation by next-generation sequencing, using a targeted panel or exome, is indicated as soon as clinical or laboratory suspicion is established.^1^ ^,^ ^2^ ^,^ ^4^

## CONCLUSIONS

Newborn screening, as a public health prevention policy, and the clinical vigilance of the pediatrician or pediatric pulmonologist, as the ability to recognize warning signs, are complementary rather than competing strategies. In isolation, neither covers the full range of IEI phenotypes.^2^ ^,^ ^3^ The most relevant goal of the investigation is early diagnosis: not merely treating an already established severe infection, but recognizing the immune defect before bronchiectasis and other sequelae are established.^5^
